# Anticancer Effect of a Novel Octahydropyrazino[2,1-a:5,4-a′]diisoquinoline Derivative and Its Synergistic Action with* Nigella sativa* in Human Gastric Cancer Cells

**DOI:** 10.1155/2017/9153403

**Published:** 2017-12-26

**Authors:** Anna Czajkowska, Agnieszka Gornowicz, Natalia Pawłowska, Robert Czarnomysy, Jolanta Nazaruk, Wojciech Szymanowski, Anna Bielawska, Krzysztof Bielawski

**Affiliations:** ^1^Department of Biotechnology, Medical University of Białystok, Kilinskiego 1, 15-089 Białystok, Poland; ^2^Department of Synthesis and Technology of Drugs, Medical University of Białystok, Kilinskiego 1, 15-089 Białystok, Poland; ^3^Herbarium of Department of Pharmacognosy, Medical University of Białystok, Kilinskiego 1, 15-089 Białystok, Poland

## Abstract

Many studies have shown that naturally occurring compounds may support prevention and treatment of various diseases, including cancer. Pharmacological investigations revealed a wide spectrum of* Nigella sativa* biological activities. Combining natural compounds together with synthetic drugs may increase the anticancer activity and limit severe side effects of such a treatment and may be an alternative to monotherapy. The aim of the study was to evaluate the cytotoxic and proapoptotic effects of a novel octahydropyrazino[2,1-a:5,4-a′]diisoquinoline derivative and its effect in combination with* Nigella sativa* seed oil or extract in human gastric cancer cells (AGS). Etoposide was used as a reference. Our studies proved that combination strategy based on novel octahydropyrazino[2,1-a:5,4-a′]diisoquinoline derivative (OM-90) with* Nigella sativa *seed oil or extract represents the strongest efficacy in AGS cancer cells as compared to monotherapy and combined treatment with* Nigella sativa *seed oil or extract together with etoposide. Such a combination also leads to the activation of mitochondrial pathway, which plays a significant role in molecular mechanism of induction of apoptosis by these compounds.

## 1. **Introduction**

Many recent studies have shown the effectiveness of combination chemotherapy in treatment for the most advanced cancers and have given an increase in response rates. The combination therapy in gastric cancer is associated with increase of survival, but also with increased toxicity. The main goal for the researches is still looking for more effective treatment choices. Antimetabolites, platinum analogs, anthracyclines, taxanes, anti-HER2, VEGF, and topoisomerase inhibitors represent the most significant classes of drugs in gastric cancer treatment [1]. The most popular agents from topoisomerase inhibitors are etoposide and irinotecan [2]. The mechanism of their action includes the inhibition of cell growth and proliferation. Etoposide is classified as Topo II inhibitor and it is also used in testicular, bladder, prostate, lung, stomach, and uterine cancer [3, 4] Chung et al. showed better results after combining 5-FU, etoposide, and cisplatin in advanced gastric cancer, but leukopenia, thrombocytopenia, and nausea/vomiting appeared [5]. Irinotecan, which is a semisynthetic derivative of camptothecin, is the most popular agent from topoisomerase inhibitors in gastric cancer treatment. Nishikawa et al. observed no survival benefit upon adding cisplatin to irinotecan after S-1 monotherapy failure in patients with advanced gastric cancer, thus giving a basis for looking novel strategies in gastric cancer treatment [6].

The novel strategy in cancer therapy is the use of chemotherapy in combination with natural compounds. The combination, which links phytotherapy with chemotherapy, may be an alternative to conventional monotherapy only based on chemotherapeutic agents and will give a possibility of reducing the doses of chemotherapeutics. The novel agents with better anticancer potential and Topo I or Topo II inhibitory function in combination with natural compounds may be strategy in gastric cancer treatment. In last years a lot of attention has been paid to* Nigella sativa* or black cumin seed. It is a great source of components with wide spectrum of biological activity. Its antineoplastic potential was also well documented [7]. Combining novel anticancer agents, which possess high anticancer activity together with* Nigella sativa* oil or extract, may be a novel strategy in cancer treatment.

**Figure 1:**
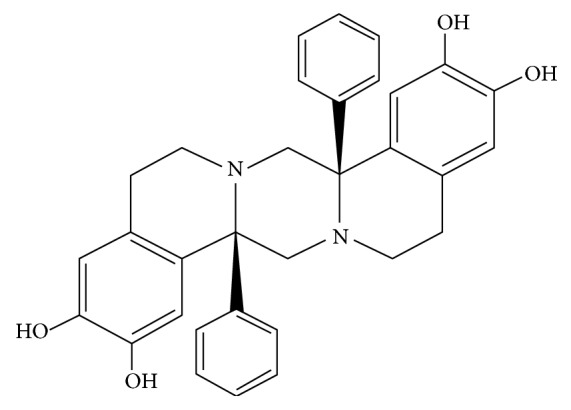
The chemical structure of octahydropyrazino[2,1-a:5,4-a′]diisoquinoline derivative (OM-90).

Recently our team has synthesized and characterized the molecular mechanism of action of novel octahydropyrazino[2,1-a:5,4-a′]diisoquinoline derivatives in estrogen receptor positive and negative breast cancer cells. These compounds were more active than etoposide and camptothecin, which are known Topo I and Topo II inhibitors in both MDA-MB-231 and MCF-7 human breast cancer cells. Flow cytometric analysis after Annexin V-FITC and propidium iodide staining also confirmed that apoptosis was the main response of human breast cancer cells to treatment of tested agents. Our results suggest that apoptosis of human breast cancer cells in the presence of analyzed compounds follows the mitochondrial pathway, with the decrease in mitochondrial membrane potential and activation of caspase 9, as well as by the external pathway with the significant increase in caspase 8 expression. Cytotoxic properties of tested compounds in cultured human breast cancer cells correlate to their ability to inhibit topoisomerase I/II [8, 9]. The most promising candidate in a group of our novel octahydropyrazino[2,1-a:5,4-a′]diisoquinoline derivatives is OM-90 (Figure 1), which possess the highest proapoptotic potential.

The aim of the study was to evaluate antiproliferative and proapoptotic properties of the combinatorial effect of* Nigella sativa* oil or extract with our novel octahydropyrazino[2,1-a:5,4-a′]diisoquinoline derivative (OM-90) in AGS-CRL-1739 gastric cancer cells compared to* Nigella sativa* oil or extract with etoposide, as well as to all of agents alone.

## 2. **Materials and Methods**

### 2.1. Chemicals and Consumables

Methanol and ethidium bromide, 3-(4,5-dimethylthiazol-2-yl)-2,5-diphenyltetrazolium bromide (MTT), were purchased from Sigma Chemical Co. (USA). Stock cultures of AGS-CRL-1739 human stomach cancer cells were purchased from the American Type Culture Collection (USA). Ham's F-12 K (Kaighn's) Medium and fetal bovine serum (FBS) used in a cell culture were products of Gibco (USA). Glutamine, penicillin, and streptomycin were obtained from Quality Biologicals Inc. (USA); [^3^H]thymidine (6.7 Ci mmol^−1^) was purchased from NEN (USA), and Scintillation Cocktail “Ultima Gold XR” from Packard (USA). Sodium dodecyl sulfate was received from Bio-Rad Laboratories (USA). Acridine orange and ethidium bromide were provided by Sigma Chemical Co. (USA). FITC Annexin V Apoptosis Detection Kit II was product of BD Pharmingen.

#### 2.1.1. *Nigella sativa* Seed Methanolic Extract

The* Nigella sativa* seeds were screened manually to remove bad ones; then they were micronized with use laboratory vibratory mill, accurately weighted (10.0 g), and extracted three times (3 × 15 minutes) with fresh portions of methanol (3 × 100 mL) in ultrasonic bath at 30°C. Obtained extracts were combined, filtered through Millex Samplicity Filters, 0.20 *μ*m (Merck, Darmstadt, Germany), and evaporated to dryness under vacuum in rotary evaporator RV 05 ST (IKA® Werke, Staufen im Breisgau, Germany). The residue was accurately weighted and dissolved in 10 mL of methanol.

#### 2.1.2. *Nigella sativa* Seed Oil

The oil from* Nigella sativa* seeds, used in this study, was the commercial one, obtained in the first cold pressing in “Dary Natury” company.

#### 2.1.3. OM-90

The octahydropyrazino[2,1-a:5,4-a′]diisoquinoline derivative (OM-90) was synthesized using previously standardized methods [9–12].

### 2.2. Cell Culture

AGS human gastric adenocarcinoma cells were maintained in base growth medium, F-12 K, supplemented with fetal bovine serum (FBS) to a final concentration of 10% and 1% antibiotics (penicillin/streptomycin). Cells were cultured in Costar flasks and grown in 5% CO_2_ at 37°C in high humid atmosphere to subconfluence (90–95%). Subconfluent cells were treated with 0.05% trypsin and 0.02% EDTA in calcium free phosphate buffered saline, counted in hemocytometer, and seeded at 5 × 10^5^ cells/well in 6-well plates (Nunc) in 2 mL of growth medium (F-12 K). Cells, which reached about 80% of confluency, were used in the present study.

### 2.3. Cell Viability Assay

Cell growth was assessed in AGS cells following treatment with single or combination therapies using MTT (3-(4,5-dimethylthiazole-2-yl)-2,5-diphenyltetrazolium bromide), according to the method of Carmichael et al. [13]. Cells were incubated with different concentrations of tested* Nigella sativa* seed oil, methanol alone,* Nigella sativa* seed methanolic extract, etoposide, and OM-90 for 24 hours to determine the IC_50_ value for each drug. The cytotoxic effect of combined therapy was also tested. Confluent cells, treated for 24 hours with studied compounds in 6-well plates, were washed three times with PBS and then incubated for 4 hours in 1 mL of MTT solution (5 mg/mL of stock in PBS) at 37°C in the atmosphere of 5% CO_2_ in an incubator. Then, supernatant was discarded and 1 mL of 0.1 mol/L HCl in absolute isopropanol was added to each well and mixed gently. Absorbance of converted dye in living cells was read at a wavelength of 570 nm using spectrophotometer (Helios Gamma UV/VIS Scanning Spectrophotometer, Unicam/ThermoFisher Scientific Inc., Waltham, MA, USA). Untreated cells were also run under identical conditions and served as control. Viability of stomach cancer cells cultured in the presence of studied oil and extract was calculated as a percent of control cells.

### 2.4. [^3^H]Thymidine Incorporation Assay

The incorporation of [^3^H]thymidine into DNA was used as a measure of cells proliferation. AGS cells were seeded in 6-well plates at a density of 5 × 10^5^ well^−1^ in a complete growth medium and grown as described above. Cells were incubated for 24 hours with various concentrations of* Nigella sativa *seed oil, methanolic extract, etoposide, and OM-90 alone as well as* Nigella sativa* oil or extract in combination with OM-90 or etoposide in 5% CO_2_ at 37°C before 0.5 *μ*Ci of [^3^H]thymidine was added to each well for 4 hours to measure the incorporation of radioactive component into the DNA. Then, the cells were harvested by trypsinization and washed several times with cold phosphate buffered saline (10 min/1500*g*) until the dpm in the washes were similar to the control reagent. Radioactivity was determined in a scintillation counter (Packard Tri-Carb Liquid Scintillation Counter 1900 TR, Perkin Elmer, Inc., Waltham, MA, USA); [^3^H]thymidine uptake was expressed as dpm well^−1^.

### 2.5. Flow Cytometry Assessment of Annexin V Binding

The effect on the induction of apoptosis was determined by Becton Dickinson FACSCanto II flow cytometer FACSCanto II (Becton Dickinson Biosciences Systems, San Jose, CA, USA), assessing the loss of asymmetry of the phospholipids on the cell membrane. Cells were trypsinized and resuspended in F12-K and then in binding buffer. Next, they were stained with FITC Annexin V and propidium iodide (PI) for 15 minutes at room temperature in the dark according to the manufacturer's instruction (FITC Annexin V Apoptosis Detection Kit II). Cells cultured in a drug-free medium were used as controls. Optimal parameter settings were found using a positive control (cells incubated with 3% formaldehyde in buffer during 30 minutes on ice). Forward scatter (FS) and side scatter (SC) signals were detected on a logarithmic scale histogram. FITC was detected in the FL1 channel (FL1 539; threshold–value 52). The results were analyzed with FACSDiva software (Becton Dickinson Biosciences Systems, San Jose, CA, USA).

### 2.6. Dual Acridine Orange/Ethidium Bromide Fluorescent Staining

To confirm the rates of apoptosis, obtained using the FITC Annexin V Apoptosis Detection Kit II, we carried out an assessment of the dual acridine orange/ethidium bromide fluorescent staining, visualized under a fluorescent microscope Nikon Eclipse Ti with an inverted camera (Nikon Instruments Inc., Melville, NY, USA). This method could be used to identify apoptosis-associated changes of cell membranes during the process of programmed cell death. It allows also accurately distinguishing cells in different stages of apoptosis. AGS-CRL-1739 human stomach cancer cells were treated with* Nigella sativa* seed oil (2.4 mg/mL),* Nigella sativa *seed methanolic extract (0.5 mg/mL), OM-90 (20 *μ*M), etoposide (20 *μ*M),* Nigella sativa* seed oil + OM-90 (2.4 mg/mL + 20 *μ*M),* Nigella sativa* seed oil + etoposide (2.4 mg/mL + 20 *μ*M),* Nigella sativa* seed methanolic extract + OM-90 (0.5 mg/mL + 20 *μ*M), and* Nigella sativa* seed methanolic extract + etoposide (0.5 mg/mL + 20 *μ*M) for 24 hours. The cell suspension (250 *μ*l) was stained with 10 *μ*l of the dye mixture (10 *μ*M acridine orange and 10 *μ*M ethidium bromide), which was prepared in PBS. Cells cultured in a drug-free medium were used as controls. The morphology of two hundred cells per sample was examined by fluorescent microscopy within 20 minutes. Obtained results were analyzed with NIS-Elements software (Nikon Instruments Inc., Melville, NY, USA).

### 2.7. Analysis of Mitochondrial Membrane Potential

Disruption of the mitochondrial membrane potential (MMP) was assessed using the lipophilic cationic probe 5,5′,6,6′-tetrachloro-1,1′,3,3′-tetraethylbenzimidazolcarbocyanine iodide (JC-1 MitoScreen kit; BD Biosciences). Briefly, unfixed cells were washed and resuspended in 10 *μ*g/mL PBS supplemented with JC-1. Cells were then incubated for 15 minutes at room temperature in the dark, washed, and resuspended in PBS for immediate BD FACSCanto II flow cytometry analysis. The percentage of cells with disrupted MMP was calculated in the FACS Diva software (both from BD Biosciences Systems, San Jose, CA, USA).

### 2.8. Statistical Analysis

Experimental data were presented as mean ± standard deviation (SD) since each experiment was repeated at least three times.

## 3. Results

Phytochemical analysis of methanolic extract obtained with ultrasound assisted extraction (MU). The chemical composition of methanolic extract obtained in ultrasonic bath was determined with chromatographic methods. The gas chromatography (GC) showed the presence of trace amount of amino acids (alanine, glycine, valine, leucine, proline, isoleucine, serine, threonine, asparagine, glutamine, and tryptophane), sugars, mono-, di-, and trisaccharides with dominating sucrose (26.07%) and raffinose (13.56%), small amount of volatile compounds (4-terpineol, thymoquinone, thymol, and longifolene), fatty acids (linoleic acid 9.41%, oleic acid 3.45%, palmitic acid 2.86%, stearic acid 0.42%, and elaidic acid 0.2%), inositols, small amounts of vitamins (pyridoxine and pantothenic acid), and phytosterols. With thin layer chromatography (TLC), flavonoid glycosides and saponins in this extract were qualitatively recognized.

**Figure 2:**
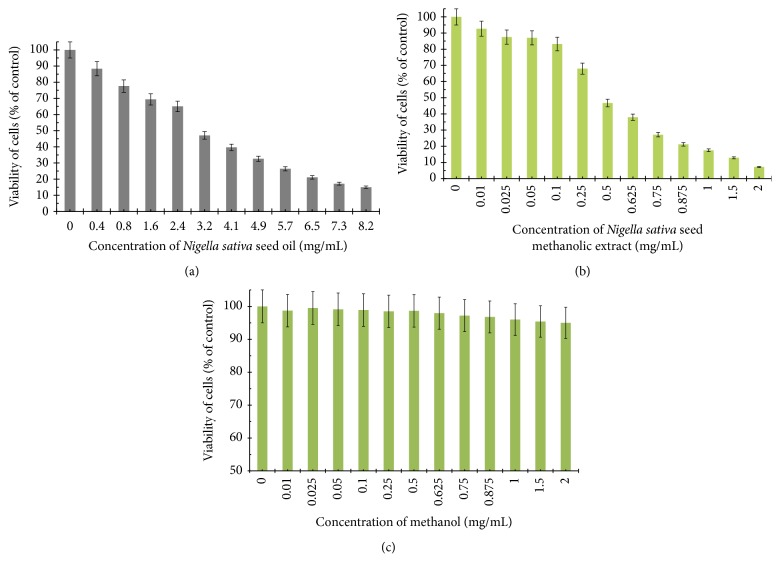
Viability of cultured AGS gastric adenocarcinoma cells exposed to various concentrations of* Nigella sativa* seed oil (a),* Nigella sativa* seed methanolic extract (b), and methanol alone (c) for 24 hours. Mean values ± SD from three independent experiments (*n* = 3) done in duplicate are presented.

**Figure 3:**
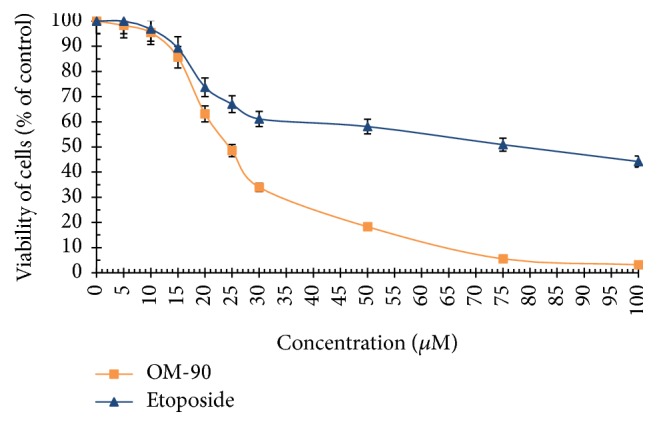
Viability of cultured AGS gastric adenocarcinoma cells treated for 24 hours with different concentrations of OM-90 and etoposide (ET). Mean values ± SD from three independent experiments (*n* = 3) done in duplicate are presented.

**Figure 4:**
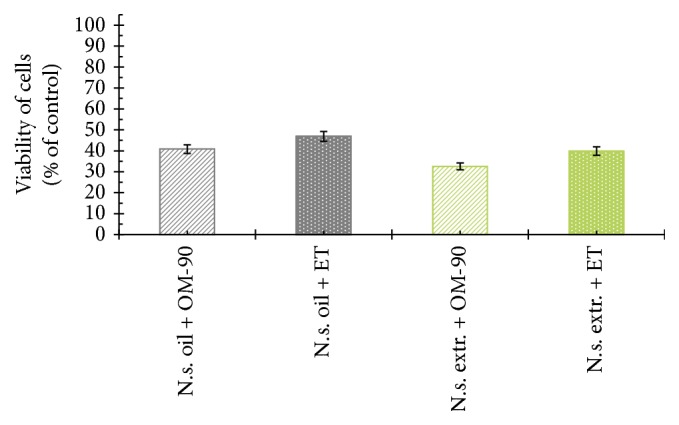
Viability of cultured AGS gastric adenocarcinoma cells treated for 24 hours with* Nigella sativa* seed oil + OM-90 (2.4 mg/mL + 20 *μ*M),* Nigella sativa* seed oil + etoposide (2.4 mg/mL + 20 *μ*M),* Nigella sativa* seed methanolic extract + OM-90 (0.5 mg/mL + 20 *μ*M), and* Nigella sativa* seed methanolic extract + etoposide (0.5 mg/mL + 20 *μ*M). Mean values ± SD from three independent experiments (*n* = 3) done in duplicate are presented.

The viability of AGS human stomach cancer cells after treatment with* Nigella sativa* seed oil, methanol alone,* Nigella sativa* seed methanolic extract, etoposide, OM-90, and combined treatment of* Nigella sativa* seed oil used together with OM-90 or etoposide, and* Nigella sativa* seed extract with OM-90 or etoposide was measured by the method of Carmichael et al. [13], using 3-(4,5-dimethylthiazol-2-yl)-2,5-diphenyltetrazolium bromide (MTT) in order to assess their cytotoxic effect. The AGS cells were exposed for 24 hours at 0.4–8.2 mg/mL concentrations of* Nigella sativa* seed oil. The tested oil decreased the number of viable cells in stomach cancer cell line in a concentration-dependent manner. The concentration of* Nigella sativa* seed oil needed to inhibit cell viability by 50% (IC_50_) in AGS human stomach cancer cells after 24 hours was found to be 3.1 mg/mL (Figure 2(a)). We also checked the effect of* Nigella sativa* seed methanolic extract on viability of gastric cancer cells (Figure 2(b)). We demonstrated that methanolic extract (0.01–2 mg/mL) exerted significant cytotoxic effect in AGS cells in a concentration-dependent manner, with IC_50_ value 0.48 mg/mL after 24 hours of incubation. We also investigated the influence of methanol alone on viability of gastric cancer cells. The results were presented in Figure 2(c). The methanol used in the highest concentration (2 mg/mL) decreased the number of viable cells to 95%. The concentration of methanol used for combined therapy was 0.5 mg/mL. We detected 98.65% of live cells and thus suggesting that methanol alone had no cytotoxic effect on viability of gastric cancer cells. OM-90 and etoposide decreased the number of viable cells in AGS gastric cancer cell line in dose-dependent manner, but OM-90 was more cytotoxic agent, with IC_50_ value of 24 ± 2 *μ*M, compared to 75 ± 2 *μ*M for etoposide. (Figure 3). A novel combination of* Nigella sativa *seed oil with OM-90 was more effective in decreasing the viability of AGS cells compared to the treatment based on* Nigella sativa* seed oil used together with etoposide (Figure 4). The combined treatment of* Nigella sativa* seed oil (2.4 mg/mL) with OM-90 (20 *μ*M) decreased the number of viable AGS cells to 40.85% and* Nigella sativa* seed oil (2.4 mg/mL) with etoposide (20 *μ*M) reduced the number of viable AGS cells to 46.87% (Figure 4). The combined treatment of* Nigella sativa* seed extract with OM-90 led to significant synergistic, cytotoxic effect compared to the* Nigella sativa* seed extract with etoposide (Figure 4).* Nigella sativa *seed extract (0.5 mg/mL) used together with OM-90 (20 *μ*M) decreased the number of viable AGS cells to 32.55%, whereas* Nigella sativa *seed extract (0.5 mg/mL) with etoposide (20 *μ*M) reduced the number of viable AGS cells to 39.85% (Figure 4).* Nigella sativa *seed extract or oil used together with OM-90 had higher cytotoxic activity in comparison to any single agent alone.

**Figure 5:**
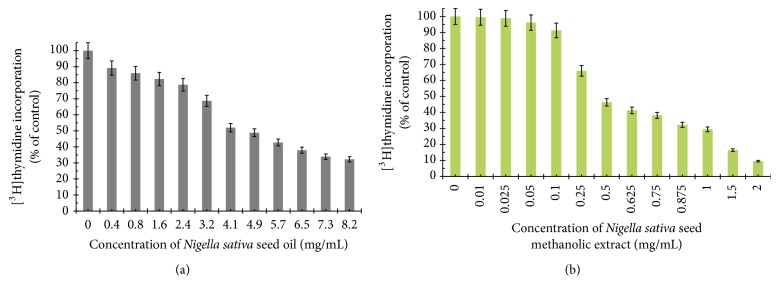
Antiproliferative effects of* Nigella sativa* seed oil (a) and methanolic extract (b) in cultured AGS gastric adenocarcinoma cells after 24 hours of incubation as measured by inhibition of [^3^H]thymidine incorporation into DNA. Mean values ± SD from three independent experiments (*n* = 3) done in duplicate are presented.

**Figure 6:**
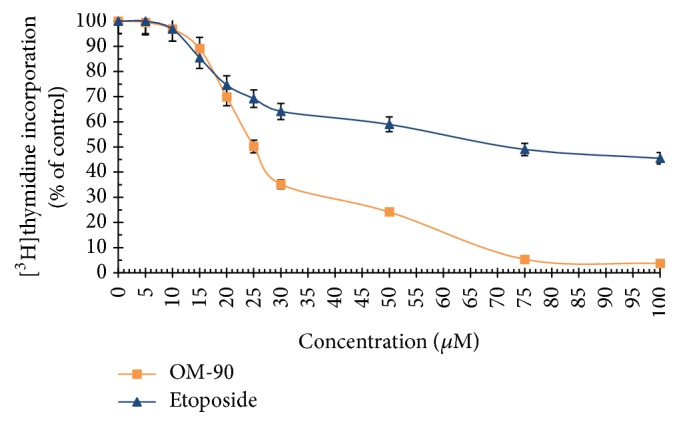
Antiproliferative effects of OM-90 and etoposide (ET) in cultured AGS gastric adenocarcinoma cells after 24 hours of incubation as measured by inhibition of [^3^H]thymidine incorporation into DNA. Mean values ± SD from three independent experiments (*n* = 3) done in duplicate are presented.

**Figure 7:**
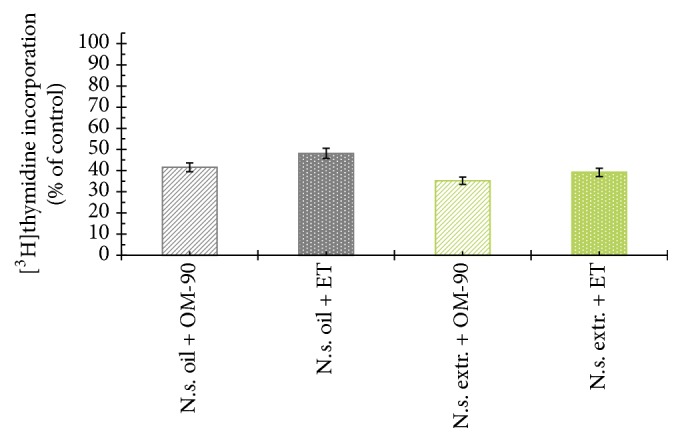
Antiproliferative effects of* Nigella sativa* seed oil + OM-90 (2.4 mg/mL + 20 *μ*M),* Nigella sativa* seed oil + etoposide (2.4 mg/mL + 20 *μ*M),* Nigella sativa* seed methanolic extract + OM-90 (0.5 mg/mL + 20 *μ*M), and* Nigella sativa* seed methanolic extract + etoposide (0.5 mg/mL + 20 *μ*M) in cultured AGS gastric adenocarcinoma cells after 24 hours of incubation as measured by inhibition of [^3^H]thymidine incorporation into DNA. Mean values ± SD from three independent experiments (*n* = 3) done in duplicate are presented.

To further investigate the possible mechanism responsible for the growth inhibitory effects, DNA synthesis was measured in the presence of* Nigella sativa* seed oil and methanolic extract (Figure 5), etoposide, and OM-90 (Figure 6), as well as combination treatment of* Nigella sativa* seed oil used together with OM-90 or etoposide and* Nigella sativa* seed extract with OM-90 or etoposide (Figure 7). All of the studied compounds showed the concentration-dependent activity, but with different potency. The concentration of* Nigella sativa* seed oil required for a 50% reduction in [^3^H]thymidine incorporation into DNA (IC_50_) in AGS human stomach cancer cells after 24 hours was found to be 4.8 mg/mL (Figure 5(a)).* Nigella sativa* seed methanolic extract also led to inhibition of the DNA biosynthesis in analyzed cells and IC_50_ value was 0.48 mg/mL after 24 hours of incubation (Figure 5(b)). Moreover, in both cases the cell viability was inhibited due to decreased cell proliferation. Furthermore, the profiles of percent cell viability and DNA synthesis obtained for* Nigella sativa* seed methanolic extract were found to be quite similar (IC_50_ values were 0.48 mg/mL and 0.46 mg/mL, resp.). The concentrations of OM-90 required to inhibit [^3^H]thymidine incorporation into DNA by 50% (IC_50_) in AGS human stomach cancer cells after 24 hours was found to be 26 ± 2 *μ*M, suggesting its higher antiproliferative potency compared to etoposide (IC_50_ = 76 ± 2 *μ*M) (Figure 6). The combined treatment of* Nigella sativa* seed oil (2.4 mg/mL) with OM-90 (20 *μ*M) was more effective in inhibition of DNA biosynthesis in AGS cells compared to the* Nigella sativa* seed oil with etoposide (Figure 7) and single treatment with* Nigella sativa *seed oil, OM-90, or etoposide.* Nigella sativa* seed oil (2.4 mg/mL) with OM-90 (20 *μ*M) reduced [^3^H]thymidine incorporation into DNA in AGS gastric cancer cells after 24 hours to 41.58% (Figure 7). It exhibited higher antiproliferative properties compared to* Nigella sativa* seed oil (2.4 mg/mL) with etoposide (20 *μ*M), which inhibited [^3^H]thymidine incorporation into DNA to 48.16% in AGS cells (Figure 7).* Nigella sativa* seed oil with etoposide strongly reduced [^3^H]thymidine incorporation into DNA in comparison to any single agent alone. The combined treatment of* Nigella sativa* seed extract with OM-90 led to stronger antiproliferative effect compared to the* Nigella sativa* seed extract with etoposide (Figure 7).* Nigella sativa* seed extract (0.5 mg/mL) in combination with OM-90 (20 *μ*M) inhibited [^3^H]thymidine incorporation into DNA to 35.21%, whereas* Nigella sativa* seed extract (0.5 mg/mL) with etoposide (20 *μ*M) reduced [^3^H]thymidine incorporation into DNA analyzed cells to 39.16% (Figure 7).

**Figure 8:**
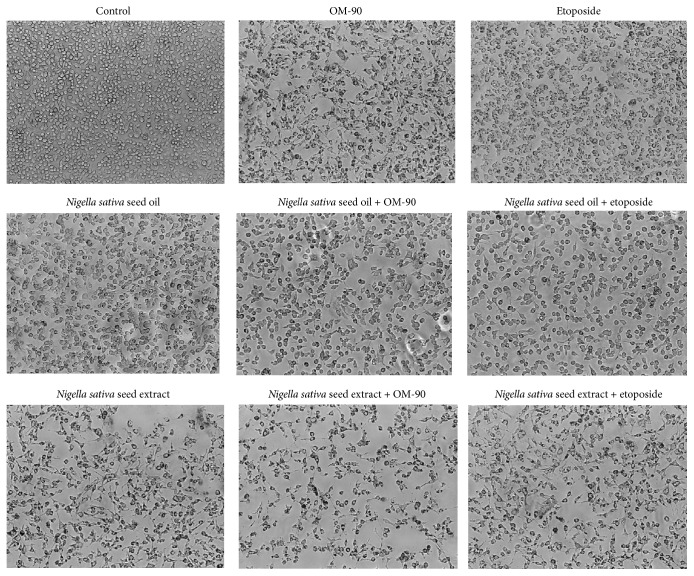
Morphological changes in cultured AGS gastric adenocarcinoma cells exposed to* Nigella sativa* seed oil (2.4 mg/mL),* Nigella sativa* seed extract (0.5 mg/mL), OM-90 (20 *μ*M), etoposide (20 *μ*M),* Nigella sativa* seed oil + OM-90 (2.4 mg/mL + 20 *μ*M),* Nigella sativa* seed oil + etoposide (2.4 mg/mL + 20 *μ*M),* Nigella sativa* seed methanolic extract + OM-90 (0.5 mg/mL + 20 *μ*M), and* Nigella sativa* seed methanolic extract + etoposide (0.5 mg/mL + 20 *μ*M) for 24 hours. Images were taken using a microscope with an inverted camera (Nikon Eclipse Ti, Nikon Instruments Inc., Melville, NY, USA) at 100x magnification.

Morphological changes in human stomach adenocarcinoma cells exposed to* Nigella sativa *seed oil and methanolic extract, etoposide, and OM-90 in monotherapy and also after combination treatment of* Nigella sativa* seed oil used together with OM-90 or etoposide, and* Nigella sativa* seed extract with OM-90 or etoposide for 24 hours are presented in Figure 8. AGS cells treated with the tested compounds exhibited altered their typical morphology and decreased cell adhesion capacity in comparison with the control. Combinations of those components led to higher alterations in the morphology of AGS cells in comparison to any agent alone in the same concentration. As shown most of the cells exposed to* Nigella sativa* seed oil with OM-90 and* Nigella sativa *seed extract with OM-90, as well as combinations of the tested oil or extract with etoposide, lost their typical morphology and appeared smaller in size. Signs of deterioration of cells also included granularity around the nucleus, detachment of the cells from the substrate, and cytoplasmic vacuolation.

**Figure 9:**
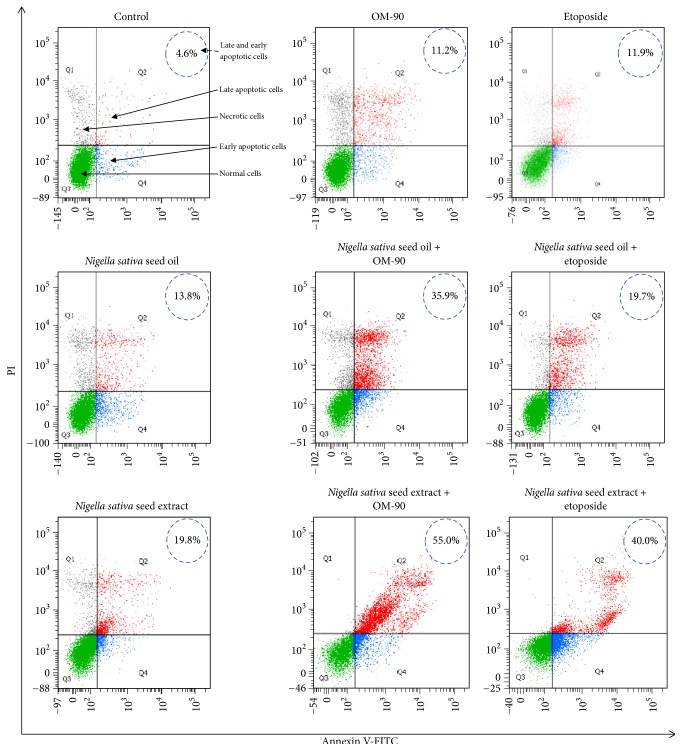
Flow cytometric analysis of cultured AGS gastric adenocarcinoma cells after incubation with* Nigella sativa* seed oil (2.4 mg/mL),* Nigella sativa* seed extract (0.5 mg/mL), OM-90 (20 *μ*M), etoposide (20 *μ*M),* Nigella sativa* seed oil + OM-90 (2.4 mg/mL + 20 *μ*M),* Nigella sativa* seed oil + etoposide (2.4 mg/mL + 20 *μ*M),* Nigella sativa* seed methanolic extract + OM-90 (0.5 mg/mL + 30 *μ*M), and* Nigella sativa* seed methanolic extract + etoposide (0.5 mg/mL + 20 *μ*M) for 24 hours and subsequent staining with Annexin V and propidium iodide (PI). Dots with Annexin V−/PI− (Q3), Annexin V+/PI− (Q4), and Annexin V+/PI+ (Q2) features represent intact, early apoptotic, and necrotic cells, respectively. Mean percentage values from three independent experiments (*n* = 3) done in duplicate are presented.

To determine the influence of analyzed compounds on induction of apoptosis in AGS human stomach cancer cells after 24 hours of treatment, we measured the cell death by flow cytometric analysis after Annexin V-FITC and propidium iodide staining (Figure 9). According to apoptosis-associated changes of cell membranes during the process of apoptosis, a clear distinction is made between normal cells, early and late apoptotic cells, and necrotic cells. Thus, cells that are considered viable or no measurable apoptosis are both Annexin V and PI negative (Annexin V−/PI−), while cells that are in early apoptosis with intact membranes are Annexin V positive and PI negative (Annexin V+/PI−), and finally cells that are in late stage of apoptosis or already dead are both Annexin V and PI positive (Annexin V+/PI+).

The incubation of AGS gastric cancer cells with* Nigella sativa* seed oil, methanolic extract, etoposide, and OM-90 in monotherapy and also combined treatment with* Nigella sativa *seed oil used together with OM-90 or etoposide and* Nigella sativa* seed extract with OM-90 or etoposide induced the visible phosphatidylserine exposure after 24 hours of treatment (Figure 9). The apoptotic effect of OM-90 was found to be quite similar than that caused by etoposide (11.2% and 11.9%, resp.).

The combined treatment based on* Nigella sativa* seed extract or oil with OM-90 led to significant effect on the cell apoptosis compared to the* Nigella sativa* seed extract, OM-90, and etoposide alone. The ratio of early and late apoptotic cells was increased after 24 hours of combined treatment with* Nigella sativa* seed extract (0.5 mg/mL) with OM-90 (20 *μ*M) in AGS cells (55.0%) compared to the treatment with* Nigella sativa* seed extract used together with etoposide (40.0%). We have found that the apoptotic effect of* Nigella sativa* seed oil with OM-90 was stronger than evoked by* Nigella sativa* seed oil with etoposide and single agents:* Nigella sativa* seed oil, OM-90, or etoposide. After combined treatment with* Nigella sativa* seed oil (2.4 mg/mL) with etoposide (20 *μ*M) in AGS cells for 24 hours the ratio of early and late apoptotic cells was increased to 19.7%. The better effect was observed after 24-hour incubation of AGS cells with* Nigella sativa *seed oil (2.4 mg/mL) and OM-90 (20 *μ*M) significantly increased the number of early and late apoptotic cells to 35.9%.

**Figure 10:**
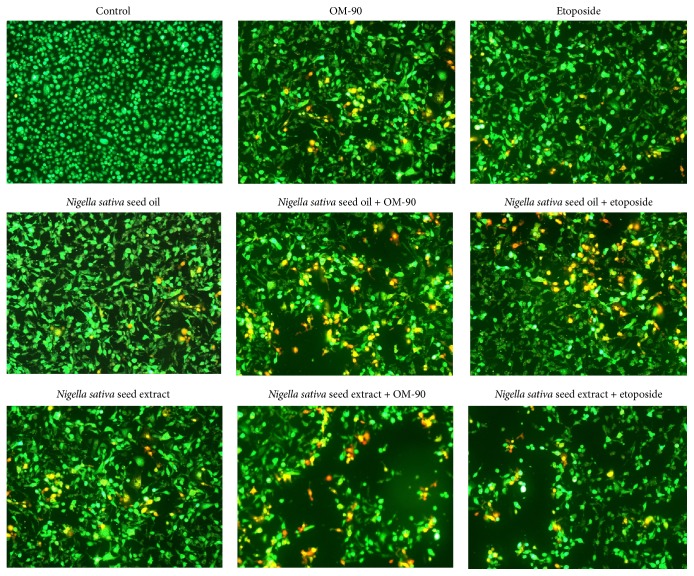
Proapoptotic properties of* Nigella sativa* seed oil (2.4 mg/mL),* Nigella sativa* seed extract (0.5 mg/mL), OM-90 (20 *μ*M), etoposide (20 *μ*M),* Nigella sativa* seed oil + OM-90 (2.4 mg/mL + 20 *μ*M),* Nigella sativa* seed oil + etoposide (2.4 mg/mL + 20 *μ*M),* Nigella sativa* seed methanolic extract + OM-90 (0.5 mg/mL + 20 *μ*M), and* Nigella sativa* seed methanolic extract + etoposide (0.5 mg/mL + 20 *μ*M) in AGS gastric adenocarcinoma cells after 24 hours of incubation, obtained using dual acridine orange/ethidium bromide (AO/EB) fluorescent staining. Images were taken using a microscope with an inverted camera (Nikon Eclipse Ti, Nikon Instruments Inc., Melville, NY, USA) at 100x magnification.

To confirm the results obtained using flow cytometry, dual acridine orange/ethidium bromide fluorescent staining was done and cell morphology was visualized in fluorescence microscope Nikon Eclipse Ti (Figure 10). These results suggest that cytotoxicity of* Nigella sativa *seed oil and methanolic extract, etoposide, and OM-90 in monotherapy and in particular combination of* Nigella sativa* seed oil used together with OM-90 or etoposide and* Nigella sativa* seed extract with OM-90 or etoposide against AGS stomach cancer cells is mainly due to inducing apoptotic cell death (as well as necrotic cell death), which is in accordance with the results observed in the above Annexin V/PI assay.

**Figure 11:**
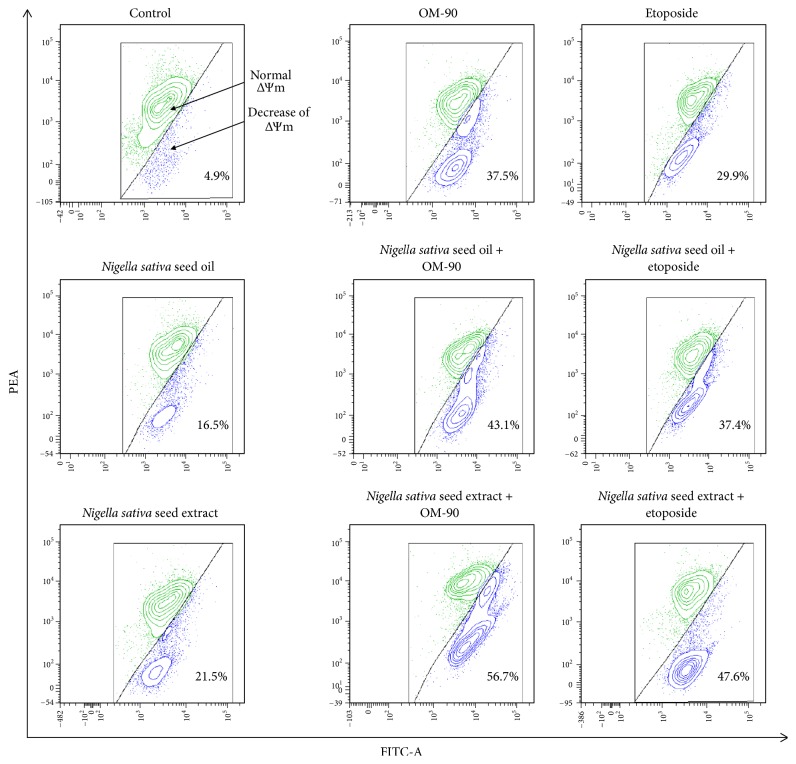
The loss of mitochondrial membrane potential of AGS gastric adenocarcinoma cells after 24 hours incubation with* Nigella sativa* seed oil (2.4 mg/mL),* Nigella sativa* seed extract (0.5 mg/mL), OM-90 (20 *μ*M), etoposide (20 *μ*M),* Nigella sativa* seed oil + OM-90 (2.4 mg/mL + 20 *μ*M),* Nigella sativa* seed oil + etoposide (2.4 mg/mL + 20 *μ*M),* Nigella sativa* seed methanolic extract + OM-90 (0.5 mg/mL + 20 *μ*M), and* Nigella sativa* seed methanolic extract + etoposide (0.5 mg/mL + 20 *μ*M) as measured JC-1 fluorescence. Mean percentage values from three independent experiments (*n* = 3) done in duplicate are presented.

In order to evaluate whether the tested compounds triggered apoptosis in gastric cancer cells, the changes of mitochondrial membrane potential (ΔΨm) were measured.  Figure 11 shows the effects of tested compounds alone and in combination on ΔΨm of AGS gastric cancer cells. The most significant decrease of ΔΨm was observed after combined treatment with* Nigella sativa* seed extract or oil together with OM-90 in comparison to combination of* Nigella sativa* extract or oil with etoposide or any agents alone (Figure 11). These results are consistent with those obtained in the Annexin V/PI assay and suggest that the apoptosis induced by the tested compounds may go through the mitochondrial pathway.

## 4. Discussion

Gastric cancer is a very aggressive tumor and represents the second leading cause of cancer mortality worldwide [14]. Systemic chemotherapy alone remains to be the mainstay strategy in cancer treatment, but resistance to chemotherapeutic agents appears and patients die in a short period of time. Many studies have shown that naturally occurring compounds may support prevention and treatment of various diseases, including cancer [15]. Pharmacological investigations revealed a wide spectrum of* Nigella sativa* biological activities including mainly antioxidant, inflammatory, antibacterial, antiviral, antiparasitic, antidiabetic, antiulcer properties [7, 16]. Currently, many studies have confirmed antitumor effect of its active ingredients against several cancer cell lines [16].* Nigella sativa* is shown to be effective against cancer in blood system, lung, kidney, liver, prostate, breast, cervix, colon, ovary, and skin cancer [7, 16–18]. There are also reports of its efficacy in the case of glioblastoma [19], fibrosarcoma [20], osteosarcoma [21], or head and neck squamous cell carcinoma [22]. Much of the biological properties of the black seed are attributed to the main constituent of its volatile oil called thymoquinone [16]. It has been shown to interact with many proteins important in signal transduction pathways, resulting in a variety of anticancer effects* in vitro* and* in vivo* [16]. Antitumor activity was also recorded for *α*-hederin, a pentacyclic triterpene saponin isolated from the seeds of* Nigella sativa* [23, 24]. Moreover, different kinds of* Nigella sativa* seed extracts [25], decoction [26], emulsion of* Nigella sativa* oil in water [27], essential oil [28], crude oil [29], and as the seeds [30, 31] were reported to possess biological activity on various types of human cancers.

Combining natural compounds together with synthetic drugs may increase the anticancer activity and limit severe side effects of such a treatment and may be an alternative to monotherapy. In recent years, researchers investigated the effect of thymoquinone with different anticancer agents such as 5-fluorouracil, topotecan, gemcitabine, oxaliplatin, docetaxel, paclitaxel, and tamoxifen in cancer cells* in vitro* [32–38].

Studies based on thymoquinone used together with topotecan proved that both drugs induced apoptosis through a p53-independent mechanism, whereas the expression of p21 was only seen in thymoquinone treatment in human colorectal cancer cells. Cell cycle arrest in the S phase was detected with each compound separately, while combined treatment only increased the production of fragmented DNA. Both compounds induced apoptosis through the extrinsic pathway after 24 hours; however, after 48 hours, the intrinsic pathway was activated by topotecan treatment only. In conclusion, thymoquinone increased the effectiveness of the chemotherapeutic reagent topotecan by inhibiting proliferation and lowering toxicity through p53- and Bax/Bcl2-independent mechanisms [34].

Banerjee et al. also underlined that combined treatment is more effective strategy in pancreatic cancer treatment.* In vitro *studies revealed that preexposure of cells with thymoquinone (25 Mmol/L) for 48 h followed by gemcitabine or oxaliplatin resulted in 60% to 80% growth inhibition compared with 15% to 25% when gemcitabine or oxaliplatin was used alone. Authors also showed that a specific target, such as NF-KB, was inactivated in animal tumors pretreated with thymoquinone followed by gemcitabine and/or oxaliplatin, thus suggesting that thymoquinone could abrogate gemcitabine or oxaliplatin-induced activation of NF-KB, resulting in the chemosensitization of pancreatic tumors to conventional therapeutics [35]. Soni et al. checked the effectiveness of dual drug-loaded paclitaxel-thymoquinone nanoparticles in MCF-7 breast cancer cells. They showed that nanoformulation composed of both these drugs had higher efficacy in breast cancer cells as compared to individual drugs alone. This formulation aimed to lower the effective anticancer dose of paclitaxel so as to alleviate the toxic effects associated with its use in clinics [39].

Surprisingly, there is insufficient number of studies concerning* Nigella sativa*'s role on gastric cancer. Lei et al. showed that thymoquinone enhanced the 5-fluorouracil-induced killing of gastric cancer cells by mediating the downregulation of the antiapoptotic protein bcl-2, the upregulation of the proapoptotic protein bax, and the activation of both caspase-3 and caspase-9. In addition results obtained* in vitro* were similar* in vivo*. It has been shown that the combined treatment of thymoquinone with 5-fluorouracil represents a significantly more effective antitumor agent than either agent alone in a xenograft tumor mouse model [32]. In our research study we checked the cytotoxic and proapoptotic potential of* Nigella sativa *seed oil or extract used together with novel octahydropyrazino[2,1-a:5,4-a′]diisoquinoline derivative (OM-90). Their effect was compared with etoposide used in combination with* Nigella sativa *seed oil or extract and all of agents alone. The strongest cytotoxic potential was observed after 24-hour incubation with* Nigella sativa* seed oil or extract together with OM-90. The weaker effect was detected after incubation with* Nigella sativa* seed oil or extract used together with etoposide, but the weakest were agents used alone. Our studies suggest that cytotoxic activity of chemotherapeutic agents (OM-90, etoposide) is enhanced by* Nigella sativa* oil or extract. Our results contradict with the study performed by Saleh et al. [40]. In our study as well as in the study performed by Saleh et al., pure thymoquinone was not used, but obtained results were different. Saleh et al. showed that the cotreatment of cancer cells (MCF-7, HepG2, U251, HeLa, and HCT116) with etoposide plus* Nigella sativa* extract did not enhance etoposide cytotoxicity [40]. The stronger, synergistic effect was observed in several previous studies, where the antitumor activity of different kinds of chemotherapeutic agents was augmented by thymoquinone in cancer cells [41–44]. Mahmoud and Torchilin showed that the therapeutic activity of doxorubicin was enhanced when it is coadministered with different* N. sativa* extracts/LE—nanoemulsion with the highest activity achieved in the case of doxorubicin—nanoemulsion preparation [45].

In our latest studies we also proved that combination treatment with chemotherapeutic agents plus monoclonal antibody against MUC1 resulted in higher cytotoxicity and better proapoptotic effect than monotherapy [46, 47], thus suggesting that combination strategy results in better efficacy than the use of chemotherapeutics alone.

In this study, we also proved that* Nigella sativa* oil or extract plus OM-90 led to the strongest apoptotic effect, which was proved by flow cytometry and fluorescence microscope. We detected the highest number of late and early apoptotic cells and typical morphological changes in analyzed cells were observed. The combined treatment based on* Nigella sativa* seed oil or extract together with OM-90 resulted in the highest decrease in mitochondrial membrane potential, thus suggesting that mitochondria are strongly involved in the induction of programmed cell death. Further studies are required to determine in detail the molecular mechanism of action.

## 5. Conclusions

The combination strategy based on novel octahydropyrazino[2,1-a:5,4-a′]diisoquinoline derivative (OM-90) together with* Nigella sativa *seed oil or extract represents the strongest efficacy in AGS cancer cells as compared to monotherapy and combined treatment of* Nigella sativa *seed oil or extract together with etoposide. Our studies suggest that mitochondria play pivotal role in the induction of apoptosis, but further studies are needed to detailed characteristic of combined action of tested compounds.
